# Supplementary material for: WordCommentsAnalyzer: A windows software tool for qualitative research
Source: F1000Res. 2018 Sep 4;7:536. Originally published 2018 May 3. [Version 2] doi: 10.12688/f1000research.14819.2 (PMC6137411; doi:10.12688/f1000research.14819.2)
Supplement: Supplementary file 3 [file f1000research-7-17614-s0002.tgz › b2a79496-4ada-47c4-9682-a99ce867836d.zip!/Eight Iranian Health Journals/Salmand_Iranian Journal of Ageing _36.docx]

© Salmand_Iranian Journal of Ageing . This work is licensed under CC BY-NC 4.0 (https://creativecommons.org/licenses/by-nc/4.0/).

Volume & Issue: دوره 4 - شماره 3

Title: بررسی مقایسه ای تصویر بدنی زنان یائسه ورزشکار و غیر ورزشکار

http://salmandj.uswr.ac.ir/article-1-315-fa.html

اهداف: هدف تحقیق حاضر، مقایسه تصویر بدنی زنان یائسه ورزشکار و غیرورزشکار بود. مواد و روش‌ها: آزمودنیهای این تحقیق را تعداد 60 نفر زن یائسه 43 تا 53 ساله تشکیل دادند که به صورت نمونه گیری خوشه ای طبقه ای از میان زنان ورزشکار و غیر ورزشکار منطقه 6 تهران انتخاب شدند. ابزار جمع آوری اطلاعات شامل پرسشنامه اطلاعات فردی محقق ساخته، همچنین پرسشنامه خود توصیفی بدنی بود که از 11 خرده مقیاس قدرت، استقامت، هماهنگی، سلامت عمومی، انعطاف پذیری، عزت نفس، لیاقت ورزشی، چربی، ظاهر بدنی، فعالیت بدنی و کل بدن تشکیل شده است. از آزمون های کولموگروف اسمیرنوف و آزمون Tمستقل برای تحلیل داده ها استفاده شد. یافته‌ها: نتایج آماری نشان داد که تصویر بدنی زنان یائسه ورزشکار در تمامی خرده مقیاسهای تصویر بدنی از جمله قدرت، استقامت، چربی بدن، ظاهر بدن، سلامت عمومی هماهنگی، انعطاف پذیری، عزت نفس، فعالیت بدنی، لیاقت ورزشی و کل بدن به طور معنی داری از زنان یائسه غیر ورزشکار بهتر بود. نتیجه‌گیری: به نظر می رسد که انجام فعالیت بدنی مستمر، اثرات مطلوبی بر شاخص روانی تصویر بدنی زنان یائسه دارد و زنان یائسه می توانند با انجام تمرینات ورزشی مستمر، اثرات مخرب روانی ناشی از یائسگی را در خود تقلیل دهند.

Title: بررسی سطح فعالیت‌های فیزیکی و شیوع بیماری‌های مزمن در سالمندان غرب تهران، 1386

http://salmandj.uswr.ac.ir/article-1-314-fa.html

اهداف: پژوهش حاضر با هدف تعیین سطح فعالیتهای فیزیکی و شیوع بیماریهای مزمن در سالمندان غرب تهران انجام گرفت. مواد و روش‌‌ها: این مطالعه از نوع مقطعی بود که بر روی 410 نفر از افراد بالای 60 سال ساکن منطقه غرب تهران انجام گرفت. روش نمونه گیری از نوع خوشه ای چند مرحله ای بود داده ها از طریق پرسشنامه محقق ساخته و مصاحبه جمع آوری شد و برای تحلیل داده از آمار توصیفی، آزمون تی–تست و ANOVA استفاده شد. یافته‌ها: فعالیتهای روزمره زندگی با متغیرهای جنس، وضعیت اقتصادی، وضعیت سلامت فعلی، سطح تحصیلات و تعداد بیماریهای مزمن ارتباط معنی داری داشت (P<0/05). فعالیتهای ابزاری زندگی نیز با متغیرهای سن، جنس، سطح تحصیلات، وضعیت اقتصادی، وضعیت سلامت فعلی، ترکیب خانواده و تعداد بیماریهای مزمن ارتباط معنی دار آماری داشت (P<0/05). همچنین 86/8درصد از نمونه ها حداقل از یک بیماری مزمن رنج می بردند. نتیجه‌گیری: مطالعه نشان داد متغیرهای مختلفی می توانند فعالیتهای فیزیکی سالمندان را تحت تاثیر قرار دهند. بیشتر سالمندان از نظر فعالیتهای فیزیکی مستقل بودند و اکثر آنها حداقل از یک بیماری مزمن رنج می بردند. لذا مراقبت دهنده های بهداشتی باید زمینه های تسهیل زندگی سالمندان را از طریق برنامه های آموزشی و نیز بررسی دوره ای وضعیت جسمانی فراهم آورند تا موجب ارتقا فعالیتهای فیزیکی در سالمندان شده و از این طریق باعث حفظ و ارتقا زندگی سالم و فعال برای آنها شوند.

Title: مطالعه منطقه‌ای، جمعیت‌شناختی و همه‌گیرشناسی برخی اختلالات روانپزشکی در شهرک اکباتان تهران

http://salmandj.uswr.ac.ir/article-1-313-fa.html

اهداف: با توجه به افزایش جمعیت سالمند در ایران و در عین حال شیوع بالای اختلالات روانپزشکی در این گروه، هدف پژوهش حاضر، بررسی ویژگی های جمعیت شناختی و همه گیر شناسی اختلالات روانپزشکی در افراد بالای 59 سال در شهرک اکباتان تهران بود. مواد و روش‌ها: نمونه پژوهش از بین تمام 1422 فرد بالای 59 سال ساکن در شهرک اکباتات تهران به طور تصادفی ساده انتخاب شد. در نهایت 204 نفر وارد طرح پژوهشی شدند و پرسشنامه های سلامت عمومی و افسردگی سالمندان را تکمیل نمودند. سپس 104 نفر به طور تصادفی انتخاب شدند و به منظور اعتبار تشخیص روانپزشکی با روش اعتبار ملاکی مصاحبه بالینی ساختاریافته، پرسشنامه تشخیصی بین المللی جامع در مورد آنها اجرا شد. یافته‌ها: شیوع طول عمر برای کل اطلاعات روانپزشکی مورد سنجش %36/9 و شیوع نقطه ای آن در بررسی یک ماهه %18/5 بود. 25/2% موارد تنها به یک اختلال روانپزشکی، %6/8 به دو اختلال و %2/9 نیز به سه اختلال روانپزشکی مبتلا بودند. شایع ترین اختلالات روانپزشکی در مطالعه حاضر به ترتیب شامل اختلالات افسردگی عمده، افسرده خوئی، وسواسی اجباری و عامل فشارزای روانی بعد از سانحه بود. نتیجه‌گیری: نتایج این مطالعه نشان داد که بعضی از اختلالات روانپزشکی در نمونه جمعیت ایرانی شیوع قابل توجه ای دارد و در عین حال نسبت به گزارشات خارجی از شیوع متفاوتی برخوردارند. به نظر می رسد یافته های فوق در برنامه ریزی مسوولان ذیربط در امر بهداشت روانی سالمندان مفید باشد.

Title: بررسی رابطه کوه‌پیمایی و سلامت جسمانی و قلبی-عروقی در سالمندان

http://salmandj.uswr.ac.ir/article-1-312-fa.html

اهداف: هدف این تحقیق بررسی اثرات کوه پیمایی بر فاکتورهای مختلف سلامتی در سالمندان بود. مواد و روش‌ها: این مطالعه یک بررسی Cross-sectional بود که در آن 43 مرد سالمند (با میانگین سنی 57/7±9.3 سال) در مناطق مرتفع شهر تهران به طور تصادفی انتخاب شدند. آزمودنی ها بر اساس نوع فعالیت به سه گروه: 1- کوه پیما (n:15)، 2- ورزشکار (n:14)، 3- غیر ورزشکار (n:14) تقسیم شدند. سپس فاکتورهای مختلف آمادگی قلبی–عروقی و جسمانی آنان (حداکثر اکسیژن مصرفی، ضربان قلب، درصد چربی، قدرت، زمان عکس العمل، فشار خون، انعطاف پذیری و تعادل) مورد ارزیابی قرار گرفت. از روش آماری آنوای یک طرفه برای تجزیه و تحلیل داده ها استفاده شد. یافته‌ها: مقایسه میانگین سه گروه در ظرفیت هوازی تفاوت معنی داری (P≤0/01) را بین آنها نشان داد که این متغیر در گروه کوهنورد نسبت به دو گروه دیگر بالاتر بود (به ترتیب %37/2 و %15/4)، به علاوه ضربان قلب استراحتی، درصد چربی بدن و همچنین زمان عکس العمل به صورت معنی داری در دو گروه فعال نسبت به گروه غیر فعال پایین تر بود (P≤0/05)، اما تفاوت بین گروه ها در دیگر فاکتورها معنی دار نبود (P≥0/05). نتیجه‌گیری: نتایج به دست آمده در رابطه با ظرفیت هوازی و درصد چربی بدن با فعالیت کوه پیمای بسیار پر اهمیت بود و تاثیر کوه پیمایی را بر فاکتورهای قلبی–عروقی نشان می دهد. بهبود نسبی ضربان قلب، قدرت، انعطاف پذیری، تعادل و زمان عکس العمل در هر دو گروه غیر ورزشکار مجددا بر تاثیر انجام فعالیت بدنی به عنوان جزء ضروری برنامه های زندگی تاکید می کند.

Title: تحرک و تعادل عملکردی در سالمندان پس از عمل جراحی تعویض کامل مفصل ران و مقایسه آن با سالمندان سالم

http://salmandj.uswr.ac.ir/article-1-311-fa.html

اهداف: هدف این مطالعه، تعیین میزان تحرک و تعادل عملکردی سالمندان پس از عمل جراحی تعویض کامل مفصل ران و مقایسه آن با سالمندان سالم بود. مواد و روش‌ها: این مطالعه مقطعی به روش نمونه گیر غیراحتمالی ساده بر روی 20 بیمار مبتلا به استئوآرتریت مفصل ران با میانگین سنی 70/9±4/2 که 6 ماه از عمل جراحی تعویض کامل مفصل ران آنها گذشته بود و 20 فرد سالم با میانگین سنی 73±5/7 به عنوان گروه کنترل با توجه به همتاسازی دو گروه انجام شد. تحرک عملکردی با آزمون تایمد آپ اند گو، تعادل عملکردی با آزمون فانکشنال ریچ و پرسشنامه تعادلی برگ و همچنین میزان استقلال در فعالیت های روزمره با پرسشنامه شاخص بارتل تغییر یافته اندازه گیری شد. داده ها یا آزمون آماری تی مستقل تحلیل شد. یافته‌ها: مقایسه بین دو گروه نشان داد که زمان انجام آزمون تایمد آپ اند گو در گروه بیمار به صورت معناداری طولانی تر از گروه کنترل بود (P<0/01)، مسافت طی شده در آزمون فانکشنال ریچ در گروه بیمار بصورت معنی داری کوتاهتر از گروه کنترل بود (P<0/01). شاخص بارتل تغییر یافته و نمره پرسشنامه تعادلی برگ در گروه بیمار نیز به صورت معناداری کمتر از گروه کنترل بود (P<0/01). نتیجه‌گیری: نتایج کاهش معنی دار تحرک و تعادل عملکردی سالمندان را پس از عمل جراحی تعویض کامل مفصل ران را در مقایسه با گروه کنترل که عمل جراحی نداشتند نشان داد.

Title: ارزش شاخص اندازه دور کمر جهت پیش‌گویی مقاومت به انسولین در مقایسه با سندرم متابولیک در سالمندان آسایشگاه کهریزک

http://salmandj.uswr.ac.ir/article-1-310-fa.html

اهداف: هدف این مطالعه، ارزیابی مقایسه ای رابطه اندازه دور کمر با سندرم متابولیک جهت پیش گویی مقاومت به انسولین در گروهی از سالمندان بود. مواد و روش‌ها: در یک مطالعه مقطعی از میان 225 سالمند 60 ساله یا بیشتر با اطلاعات بالینی کامل 94 نفر (42 مرد و 52 زن)، که قادر به امضا فرم رضایتناه نمره بالاتر از 20 در آزمون روانی مختصر بودند و تمایل شرکت در مطالعه را داشتند، وارد مطالعه شدند. حساسیت به انسولین با استفاده از نمایه ارزیابی مدل هوموستاز تعیین شد. مقاومت به انسولین به صورت بالاترین چارک این نمایه تعریف شد. یافته‌ها: در هر دو جنس، دور کمر و نمایه ارزیابی مدل هوموستاز رابطه قوی مثبتی با هم داشتند. دور کمر مطلوب برای مردان 94/5 سانتی‌متر و برای زنان 90/5 سانتی‌متر بود. حساسیت و ویژگی بالای دور کمر در مردان (به ترتیب 0/80 و 0/84) نشان دهنده توانایی بالای این شاخص در تشخیص مبتلایان به مقاومت به انسولین بوده که می توان تمامی مردان غیر مبتلا به مقاومت به انسولین را به طور صحیح شناسایی کند. نتیجه‌گیری: در این مطالعه، مقدار مرزی 94/5 سانتی‌متر به عنوان شاخص مقاومت به انسولین در مردان تعیین شد. به نظر می رسد که تعیین دور کمر، مستلزم انجام مطالعات بیشتر بر روی جمعیتهای بزرگتر از افراد مبتلا به سندرم متابولیک است.

Title: بررسی تکرارپذیری عملکرد تعادلی تکلیف منفرد و دوگانه با استفاده از شاخص‌های صفحه نیرو در افراد سالمند سالم

http://salmandj.uswr.ac.ir/article-1-309-fa.html

اهداف: هدف مطالعه بررسی تکرارپذیری شاخص های صفحه نیرو در هر دو حالت اجرای تکلیف تعادلی منفرد و دوگانه، و در حالات مختلف دشواری پاسچرال بود. مواد و روش‌ها: افراد سالمند بالای 65 سال و سالم، در دوجلسه با فاصله یک هفته مورد ارزیابی تعادلی قرار گرفتند. با ترتیبی تصادفی از ترکیب سه سطح دشواری پاسچرال (ایستادن در شرایط چشم باز بدون واسطه کفی، چشم بسته بدون واسطه کفی و چشم بسته با واسطه کفی) و شرایط فعالیت شناختی (ایستادن بدون/همراه با انجام فعالیت شناختی شمارش معکوس) سیگنال مرکز فشار ثبت و شاخص های صفحه نیرو محاسبه گردید. ضریب تکرارپذیری، خطای استاندارد میانگین، و ضریب تغییرات برای تمامی حالات و متغیرها محاسبه گردید. یافته‌ها: در وضعیت تکلیف منفرد مساحت نوسان مرکز فشار، انحراف معیار شدت در هر دو جهت، و انحراف معیار سرعت طرفی، در تمامی سطوح دشواری تکلیف پاسچرال از تکرارپذیری بالا تا بسیار بالا برخوردار بودند. در وضعیت تکلیف دوگانه سرعت میانگین کلی و انحراف معیار سرعت در هر دو جهت در تمامی سطوح دشواری تکلیف پاسچرال از تکرارپذیری بالا تا بسیار بالا برخوردار بودند. نتیجه‌گیری: به طور کلی، پروتکل سنجش تعادل مورد استفاده در مطالعه حاضر، در هر دو حالت تکلیف منفرد و تکلیف دوگانه تکرارپذیری نسبی و مطلق مناسبی در افراد سالمند سالم نشان داده است.

Title: آیا ارزیابی مختصر تغذیه‌ای، شاخص پیش آگهی خوبی برای فعالیت روزانه سالمندان است؟

http://salmandj.uswr.ac.ir/article-1-308-fa.html

اهداف: اهداف مطالعه حاضر، ارزیابی ارتباط بین ارزیابی مختصر تغذیه با فعالیت روزانه برای پیش آگهی عملکرد خوب و همچنین بررسی ارزیابی مختصر تغذیه به عنوان ابزاری برای فعالیت روزانه در سالمندان ساکن خانه سالمندان بود. مواد و روش‌ها: در یک مطالعه مقطعی از میان 1200 پرونده ثبت شده تعداد 247 سالمند 60 ساله و بالاتر، پرسشنامه ارزیابی مختصر تغذیه و شاخص بارتل را تکمیل کردند. ارتباط بین متغیرهای مورد مطالعه با استفاده از آزمون همبستگی پیرسون و اسپیرمن بررسی شد. برای تایید این ارتباط از مدل رگرسیون مشابه سازی شده استفاده شد. یافته‌ها: همبستگی بین امتیاز ارزیابی منحصر تغذیه و بارتل معنادار بود (P<0/001، r=0/26). در مدل مشابه سازی شده بر اساس سن و جنس، شاخص بارتل بطور معناداری با سن (P<0/006)، نمایه توده بدن (P<0/009)، و ارزیابی مختصر تغذیه (P<0/001) مرتبط بود. نتیجه‌گیری: شاخص ارزیابی مختصر تغذیه می توان ابزار پیش آگهی خوب فعالیت روزانه در سالمندان ساکن خانه سالمندان باشد.
